# Sensitivity and specificity of the Cobas Liat CT/NG/MG nucleic acid test in a clinical laboratory setting and point-of-care location

**DOI:** 10.1128/jcm.00706-25

**Published:** 2025-10-29

**Authors:** B. Van Der Pol, R. Arcenas, C. Boraas, S. Chavoustie, L. L. Crane, N. d'Empaire, A. C. Ermel, G. Harnett, F. Hinestrosa, S. House, R. A. Lillis, J. Miller, A. Mills, R. Poblete, S. A. Young, on behalf of the Liat CT/NG/MG Clinical Trial Study Group, Diana Orta

**Affiliations:** 1 BioCollections Worldwide, Inc., Miami, USA; 2 Indiana University School of Medicine, Indianapolis, USA; 3 North Jersey Community Research Initiative, Newark, USA; 4 Orlando Immunology Center, Orlando, USA; 5 University of Alabama, Birmingham, USA; 6 No Resistance.com, Birmingham, USA; 7 Washington University School of Medicine, St. Louis, USA; 1 University of Alabama at Birmingham Heersink School of Medicine 9967, Birmingham, Alabama, USA; 2 Roche Molecular Systems 53527 , Pleasanton, California, USA; 3 Planned Parenthood North Central States 590900 , Saint Paul, Minnesota, USA; 4 Segal Trials North Miami Office, North Miami, Florida, USA; 5 Planned Parenthood Gulf Coast 168711 , Houston, Texas, USA; 6 BioCollections Worldwide, Inc., Miami, Florida, USA; 7 Indiana University School of Medicine 12250, Indianapolis, Indiana, USA; 8 No Resistance Consulting Group, LLC, Birmingham, Alabama, USA; 9 Orlando Immunology Center, Orlando, Florida, USA; 10 Washington University School of Medicine 12275, St. Louis, Missouri, USA; 11 Henry Ford Hospital 24016, Detroit, Michigan, USA; 12 Mills Clinical Research, Los Angeles, California, USA; 13 North Jersey Community Research Initiative 649538 , Newark, New Jersey, USA; 14 TriCore Reference Laboratories 159777 , Albuquerque, New Mexico, USA; 15 Louisiana State University Health Sciences Center 12258, New Orleans, Louisiana, USA; Marquette University, Milwaukee, Wisconsin, USA

**Keywords:** *Chlamydia trachomatis*, Cobas Liat, *Mycoplasma genitalium*, *Neisseria gonorrhoeae*, nucleic acid testing, point-of-care

## Abstract

Sexually transmitted infections, e.g., *Chlamydia trachomatis* (CT), *Neisseria gonorrhoeae* (NG), and *Mycoplasma genitalium* (MG), present with similar signs/symptoms and are often treated empirically due to delays in receiving diagnostic test results. This study evaluated the clinical performance of the Cobas Liat CT/NG/MG nucleic acid amplification test (NAAT) in detecting these pathogens in a point-of-care (POC) setting. This non-interventional, multicenter study recruited symptomatic/asymptomatic participants from 13 geographically diverse POC sites across the USA. Prospective clinician- and self-collected specimens (urine/vaginal swabs) were used to determine the sensitivity and specificity of the Cobas Liat CT/NG/MG test relative to the composite reference standard (CRS), determined using a combination of results from three FDA-approved NAATs and one laboratory-developed test. Among 4,800 evaluable participants, 40.4% (*n* = 1,941) were symptomatic, and 51.9% (*n* = 2,489) were female at birth. The Cobas Liat CT/NG/MG test demonstrated good clinical performance across all specimen types regardless of symptom status, with specificity >97% for each analyte, and sensitivity ≥92%, except female urine (≥CT 87%, NG ≥ 83%, MG ≥ 77%). The test was considered easy to use, and no statistically significant difference in performance results was observed between trained/untrained users. The Cobas Liat CT/NG/MG test demonstrated good clinical performance, with high sensitivity and specificity for CT/NG/MG detection, regardless of symptom status. The assay provides a short turn-around time (approximately 20 min) with centralized testing laboratory accuracy at the POC for self- and clinician-collected samples. POC testing can facilitate rapid pathogen identification and accurate treatment, thereby mitigating the need for empiric treatment.

**IMPORTANCE:**

Nucleic acid amplification tests are the preferred method for diagnosing CT and NG infections and are the only reliable tests for MG; however, delays in receiving results can lead to empiric treatment, potentially causing misdiagnosis and overtreatment of STIs. Point-of-care testing could mitigate these issues by enabling rapid pathogen identification and treatment, but no rapid POC tests are currently available for the detection of CT, NG, and MG despite the similarity of symptoms, and these three pathogens being responsible for the majority of symptomatic STIs in some settings. Our findings suggest that the Cobas Liat CT/NG/MG assay may help to reduce reliance on empiric treatment of symptoms and minimize resulting return visits for unresolved infections. Use of the Cobas Liat CT/NG/MG assay may also result in improved patient outcomes, help realize population benefits by reducing the duration of infection and potentially transmission, and reduce healthcare costs.

## INTRODUCTION

Sexually transmitted infections (STIs) represent a major public health challenge, with more than 1 million STIs acquired daily worldwide (1). *Chlamydia trachomatis* (CT) and *Neisseria gonorrhoeae* (NG) are among the most common curable STIs, with estimated global annual incidences of 127.2 million and 86.9 million cases, respectively (2). In the USA in 2022, 1.65 million cases of CT and 0.65 million cases of NG were reported (3).

Infection with CT or NG can cause various urogenital conditions, including cervicitis, urethritis, vaginitis, and proctitis (2). More recently, *Mycoplasma genitalium* (MG) has emerged as an important cause of urogenital infections in both men and women and is responsible for 15%–20% of non‐gonococcal urethritis (NGU), 20%–25% of non-chlamydial NGU, and 40% of persistent or recurrent urethritis in men in addition to being detected in up to 30% of women with clinical cervicitis (4).

If left untreated, these STIs may lead to clinical sequelae, including chronic pelvic pain, pelvic inflammatory disease (PID), infertility, ectopic pregnancy, neonatal complications, and epididymitis; they are also implicated in an increased risk of HIV infection (2, 5, 6). Many individuals with CT, NG, and/or MG infections are asymptomatic but may still experience the long-term sequelae associated with STIs in addition to unwittingly transmitting these STIs to their sexual partners (2, 6–8).

CT, NG, and MG present with similar clinical signs and symptoms and can only be diagnosed with etiological or microbiological testing. The US Centers for Disease Control and Prevention (CDC) recommends annual routine CT and NG screening for all sexually active females aged <25 years (6). CT screening of sexually active young males is recommended in clinical settings serving populations of young men with a high prevalence of chlamydial infections, and CT and NG screening is recommended at least annually in young males who have sex with males (6). Routine MG testing is not currently recommended by the CDC for primary cases of NGU in men or cervicitis in women but is recommended in men with recurrent NGU and women with recurrent cervicitis or PID. Additionally, testing of sexual partners of patients with symptomatic MG infection should be considered, except in settings that use expedited partner therapy—the clinical practice of treating the sexual partners of persons who are unable or unlikely to seek timely treatment (6, 9). Meanwhile, European (2021) and British (10) guidelines both recommend MG testing in symptomatic patients and post-treatment retesting to ensure microbiological cure (10, 11).

Nucleic acid amplification test (NAAT) technologies are the CDC-recommended standard for the detection of both CT and NG infections (12, 13) and are the only reliable test type for MG detection (6, 7). However, delays in receiving diagnostic results from laboratory-based NAATs mean that many patients are receiving empiric treatment before laboratory results are available (14). This strategy of syndromic case management, whereby clinical management decisions are made based on a patient’s symptoms and signs, is recommended for resource-limited settings but is also widely used in well-resourced settings (12, 13, 15, 16). However, this approach can lead to misdiagnosis, resulting in overtreatment, inappropriate prescriptions, and missed treatment (13, 15).

The use of NAATs in centralized laboratories has given clinicians the option to have highly sensitive and specific testing for their patients. However, samples must be transported from the clinic to a centralized or reference laboratory for testing, with results available a few days later. Inappropriate treatment increases the risks of onward transmission and results in poor clinical outcomes (e.g., development of PID), while overtreatment is a key concern for antimicrobial stewardship and contributes to antimicrobial resistance (13). From a patient’s perspective, delayed treatment and the necessity for follow-up appointments can lead to loss to follow-up, increased anxiety levels, and concerns about the stigma associated with STI testing (17, 18). In the post-COVID era, there is increasing patient expectation for point-of-care (POC) testing (19). All of these issues may be mitigated with clinical POC testing that facilitates rapid pathogen identification and appropriate treatment administration (17, 18, 20, 21).

The Cobas Liat CT/NG/MG test (Roche Molecular Systems, Pleasanton, CA) for use on the Cobas Liat system (an automated nucleic acid test instrument) uses real-time polymerase chain reaction (PCR) technology to rapidly (approximately 20 minutes) (22) detect and differentiate between CT, NG, and MG in easy-to-collect urogenital specimens (either urine or vaginal swabs). The Cobas Liat CT/NG/MG assay is not cleared by the US Food and Drug Administration (FDA) for female urine specimen type. The Cobas Liat CT/NG/MG assay is cleared for all intended specimen types for CE-marked countries. In this prospective, multicenter study, we evaluated the clinical performance of the Cobas Liat CT/NG/MG test in both symptomatic and asymptomatic men and women from geographically diverse sites across the USA.

## MATERIALS AND METHODS

### Patient population

In this multicenter study, we recruited participants from family planning, emergency department, obstetrics and gynecology, and STI clinics from geographically diverse sites across the USA (Fig. S1). We invited individuals ≥14 years of age, who were assigned male or female at birth (hereafter referred to as male and female), and who reported sexual activity with a new partner or multiple partners within the previous 3 months to participate. A full list of exclusion criteria is available in (Table S1).

We collected clinical and laboratory information for all participants who were classified as symptomatic if they self-reported one or more of the following symptoms: dysuria; urinary urgency/frequency; urogenital discharge; urogenital pain, discomfort, or irritation; pain, discomfort, or abnormal bleeding related to sexual intercourse; intermenstrual bleeding with or without pain; and testicular or scrotal swelling/tenderness. Assignment of symptomatic status was also based on clinical signs observed by clinicians.

### Study ethics

We conducted the study in compliance with the International Conference on Harmonisation Good Clinical Practice Guidelines and regulations of the FDA. Institutional review board approval was obtained for each participating study site before study start. All participants provided written informed consent.

### Specimens

Male and female participants provided first-catch urine samples, which were aliquoted by test operators into the respective collection devices for Cobas Liat and comparator reference testing, as detailed in Table S2.

In addition, female participants were randomized into two separate arms and provided four vaginal swabs. Participants in both arms provided three clinician-collected vaginal swabs that were analyzed by the comparator reference tests (Table S2), with the order of collection rotated between collection devices. The fourth vaginal swab was collected in Cobas PCR Media (Cobas Liat CT/NG/MG test) by a clinician in the first group and by the patient in the second arm.

Use of archived specimens from a previous study (23) was necessary due to the expected low prevalence of NG in the prospectively collected specimens; the archived specimens included an equal number of NG-positive and NG-negative male urine, female urine, and vaginal swabs samples.

### Sample testing

All study sites performed both prospective specimen collection and testing following the respective assay’s instructions for use from the manufacturer (including information for optimal collection and storage). Samples were analyzed at the clinical site using the Cobas Liat CT/NG/MG test and then shipped to two reference laboratory sites for comparator testing. For prospective specimens, the composite reference standard (CRS) designations of “positive” or “negative” for each individual analyte (CT, NG, and MG) were based on combined results from three FDA-cleared NAATs and one laboratory-developed test using clinician-collected vaginal swab specimen from female participants and urine specimen from male participants (Table S2). The composite comparator result was defined as the concordant result from two comparator assays (NAAT1 and NAAT2). In case of discordance between the two comparator assays, we tested the sample by a third method (NAAT3) and the result of that test determined the composite comparator status (Table S3).

### Operator training

The Cobas Liat CT/NG/MG test and comparator reference tests were performed at two laboratory sites by operators who were familiar with conducting NAATs (laboratorians). In addition, the Cobas Liat CT/NG/MG test was performed at POC sites by operators considered to be representative of typical POC operators (e.g., nurses and medical assistants), having read the Cobas Liat system user materials prior to study testing; no other instrument training was provided (non-laboratorians). Definition of laboratorians/non-laboratorians was based on Yes/No responses to the question “Prior to working with this device, have you had any training or hands-on experience conducting laboratory testing as a trained professional?” Non-laboratorians met the requirements to perform Clinical Laboratory Improvement Amendments (CLIA) waived testing (24).

Upon completion of the study, the non-laboratorians completed ease-of-use questionnaires. Analyses were also performed to compare the sensitivities and specificities of the Cobas Liat CT/NG/MG test when conducted by the non-laboratorians vs laboratorians.

### Data analysis and interpretation of results

We interpreted test results for each assay according to the manufacturer’s instructions. Results were considered invalid if there were unacceptable or missing external control run results, protocol deviations, incidents (e.g., external power failure), or if data were generated during troubleshooting of the instrument system or assay. The clinical performance of the Cobas Liat CT/NG/MG test was determined by comparing test results to the CRS.

The sensitivity and specificity, with two-sided 95% confidence intervals (*CIs*), were calculated overall, according to symptom status for each sex, and by specimen type, and were compared with the CRS. The data presented for NG represent the overall findings for the prospectively collected and archived specimens. Significance was defined using Fisher’s *z*-test with alpha = 0.05. Statistical analyses were performed using SAS/STAT software.

Pairwise agreement between the Cobas Liat CT/NG/MG test and NAAT1 and NAAT2 assays was investigated by calculating positive percent agreement (PPA), negative percent agreement (NPA), and overall percent agreement (OPA) values for each analyte and specimen type.

## RESULTS

### Study population

**Fig 1:**
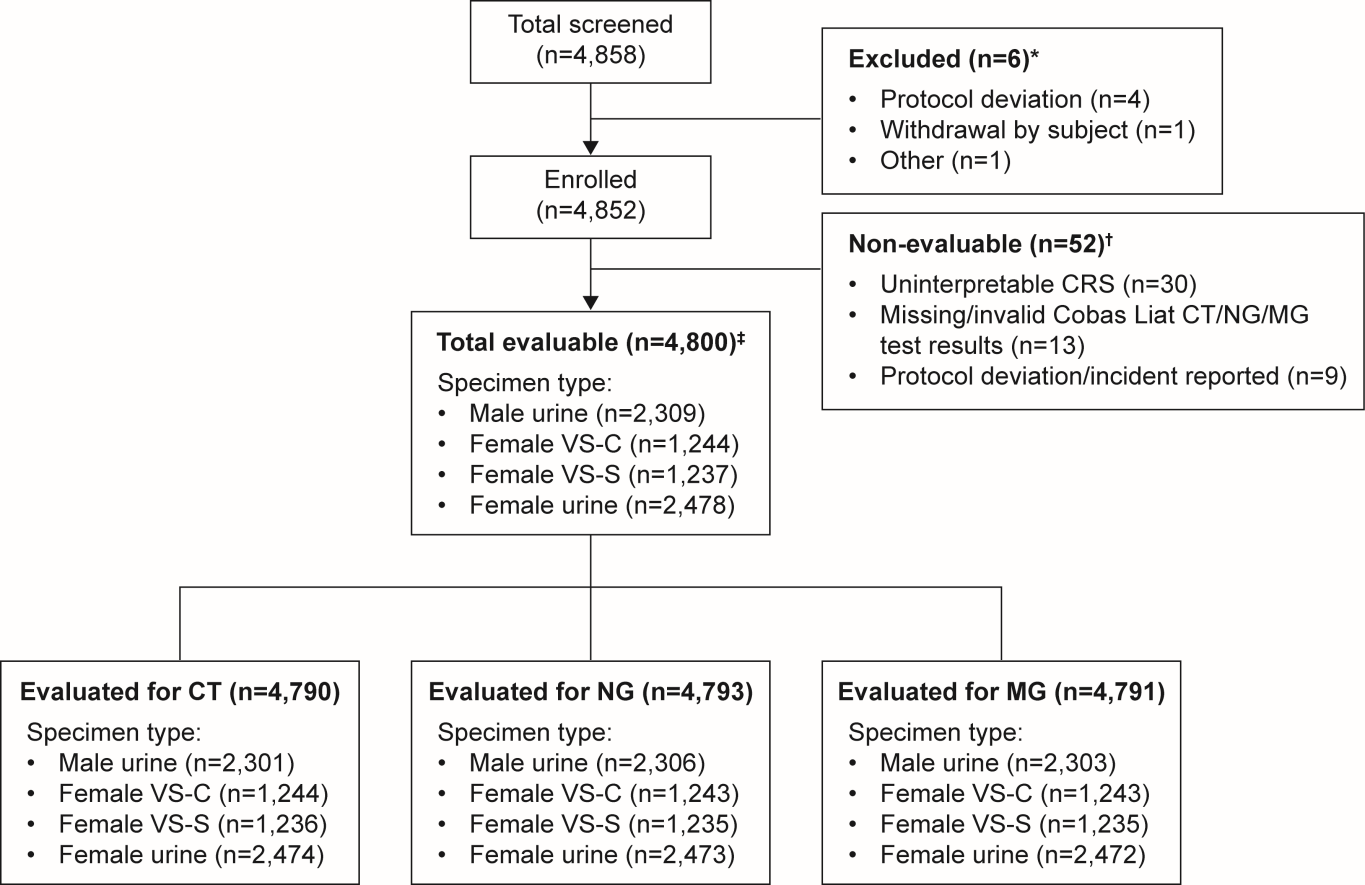
Subject and sample disposition. *Participants who did not meet the study criteria were excluded from all analyses. ^†^Non-evaluable participants were those with invalid Cobas Liat CT/NG/MG test results across all specimen types and/or with an “uninterpretable” CRS. ^‡^Participants with a positive or negative CRS result and a valid test result with the Cobas Liat CT/NG/MG test were considered evaluable and included in the final analysis. CRS = composite reference standard. CT = *Chlamydia trachomatis*. MG = *Mycoplasma genitalium*. NG = *Neisseria gonorrhoeae*. VS-C = clinician-collected vaginal swab. VS-S = self-collected vaginal swab.

A total of 4,858 individuals were screened, of whom 4,852 were eligible for inclusion; 6 (0.1%) individuals were excluded due to withdrawn consent (*n* = 1), protocol deviations (*n* = 4), or other reasons (*n* = 1) (Fig. 1). Among the 4,852 eligible participants, 52 were classified as non-evaluable and excluded from all statistical analyses; reasons for exclusion are provided in Fig. 1; Table S4.

Of the 4,800 evaluable participants, 51.9% (*n* = 2,489) and 48.1% (*n* = 2,311) were assigned female and male at birth, respectively; 40.4% (*n* = 1,941) were symptomatic; and 59.6% (*n* = 2,859) were asymptomatic (Table 1). Most participants identified as a cisgender woman (50.9%; *n* = 2,443) or cisgender man (46.6%; *n* = 2,238); 0.9% (*n* = 43) and 0.3% (*n* = 12) identified as transmasculine or transfeminine, respectively, and 1.2% (*n* = 58) identified as non-binary, gender-queer, or not exclusively man or woman (Table 1).

**TABLE 1:** Demographics and baseline characteristics^*a*^

Characteristic	Evaluable participants (*N* = 4,800)
Age, years	
Mean ± SD	37.9 ± 13.51
Median	35.0
Range	15.0–81.0
Sex assigned at birth	
Female	2,489 (51.85)
Male	2,311 (48.15)
Current gender identity	
Cisgender woman	2,443 (50.90)
Cisgender man	2,238 (46.63)
Transfeminine	43 (0.90)
Transmasculine	12 (0.25)
Non-binary, gender-queer, or not exclusively man or woman	58 (1.21)
Other	1 (0.02)
Prefer not to answer	5 (0.10)
Ethnicity	
Hispanic or Latino	1,136 (23.67)
Not Hispanic or Latino	3,612 (75.25)
Not reported	34 (0.71)
Unknown	18 (0.38)
Race	
American Indian or Alaska Native	34 (0.71)
Asian	89 (1.85)
Black or African American	2,465 (51.35)
Native Hawaiian or other Pacific Islander	13 (0.27)
White	1,916 (39.92)
Not reported	103 (2.15)
Other	180 (3.75)
Symptom status	
Symptomatic	1,941 (40.44)
Asymptomatic	2,859 (59.56)
Is the subject pregnant?	
Yes	41 (0.85)
No	2,462 (51.29)
Unknown	67 (1.40)
Not applicable	2,230 (46.46)
Pregnancy confirmatory test^*b*^	
Home pregnancy test	7 (17.1)
Clinical urine test	21 (51.1)
Blood test	5 (12.2)
Other^*c*^	8 (19.5)

^*a*^ Participants with a positive or negative composite reference standard and corresponding final valid Cobas Liat CT/NG/MG test result for either CT, NG, or MG were considered evaluable and included in this summary table. Data are *n* (%) unless otherwise indicated. CT = *Chlamydia trachomatis*. MG = *Mycoplasma genitalium*. NG = *Neisseria gonorrhoeae*. SD = standard deviation.

^*b*^ Percentage calculated from the number of pregnant participants (*n* = 41).

^*c*^ Other includes combinations of confirmatory tests: four clinical urine and blood tests; two home pregnancy tests, clinical urine, and blood tests; one home pregnancy test and blood test; and one home pregnancy test and other test.

Archived specimens used for NG testing included 163 male urine samples, 53 clinician-collected vaginal samples, 37 self-collected vaginal samples, and 90 female urine samples. The demographic characteristics of participants contributing archived specimens were comparable to those of the participants contributing prospective specimens, allowing the amalgamation of archived and prospectively collected data (data not shown).

### Infection status as defined by the CRS

The proportion of participants with infections as defined by the CRS is presented by symptom status and specimen type for CT (Table 2), NG (Table 3), and MG (Table 4). Positivity based on CRS status was generally more common among symptomatic vs asymptomatic participants (e.g., 6.9% and 3.7%, respectively, for CT positivity in male urine specimen; Table 2); however, positivity was marginally lower for symptomatic compared with asymptomatic participants using clinician-collected vaginal swabs for NG (3.3% and 3.4%, respectively; Table 3) and self-collected vaginal swabs for MG (10.5% and 11.8%, respectively; Table 4).

**TABLE 2:** Sensitivity and specificity of the Cobas Liat CT/NG/MG nucleic acid test for detection of CT by specimen type and symptom status (prospectively collected specimen)^*a*^

Specimen type	Symptom status	Total (*N*)	Positive CRS	CT sensitivity		CT specificity	
			% (*n*/*N*)	% (*n*/*N*)	95%	%, (*n*/*N*)	95%
Male urine	Symptomatic	808	6.9 (56/808)	98.2 (55/56)	(90.6, 99.7)	99.9 (751/752)	(99.3, 100)
	Asymptomatic	1,488	3.7 (55/1,488)	96.4 (53/55)	(87.7, 99.0)	99.9 (1,432/1,433)	(99.6, 100)
	Overall	2,296	4.8 (111/2,296)	97.3 (108/111)	(92.4, 99.1)	99.9 (2,183/2,185)	(99.7, 100)
Female VS-C	Symptomatic	553	5.6 (31/553)	96.8 (30/31)	(83.8, 99.4)	99.6 (520/522)	(98.6, 99.9)
	Asymptomatic	686	2.8 (19/686)	94.7 (18/19)	(75.4, 99.1)	100 (667/667)	(99.4, 100)
	Overall	1,239	4.0 (50/1,239)	96.0 (48/50)	(86.5, 98.9)	99.8 (1,187/1,189)	(99.4, 100)
Female VS-S	Symptomatic	563	5.3 (30/563)	100 (30/30)	(88.6, 100)	99v8 (532/533)	(98.9, 100)
	Asymptomatic	671	4.3 (29/671)	100 (29/29)	(88.3, 100)	99.7 (640/642)	(98.9, 99.9)
	Overall	1,234	4.8 (59/1,234)	100 (59/59)	(93.9, 100)	99.7 (1,172/1,175)	(99.3, 99.9)
Female VS**^*b*^**	Symptomatic	1,116	5.5 (61/1,116)	98.4 (60/61)	(91.3, 99.7)	99.7 (1,052/1,055)	(99.2, 99.9)
	Asymptomatic	1,357	3.5 (48/1,357)	97.9 (47/48)	(89.1, 99.6)	99.8 (1,307/1,309)	(99.4, 100)
	Overall	2,473	4.4 (109/2,473)	98.2 (107/109)	(93.6, 99.5)	99.8 (2,359/2,364)	(99.5, 99.9)
Female urine	Symptomatic	1,116	5.5 (61/1,116)	86.9 (53/61)	(76.2, 93.2)	99.8 (1,053/1,055)	(99.3, 99.9)
	Asymptomatic	1,354	3.5 (47/1,354)	87.2 (41/47)	(74.8, 94.0)	99.8 (1,304/1,307)	(99.3, 99.9)
	Overall	2,470	4.4 (108/2,470)	87.0 (94/108)	(79.4, 92.1)	99.8 (2,357/2,362)	(99.5, 99.9)

^*a*^ Positive or negative CRS results were derived from combined results from the reference NAATs; these included NAAT results from urine specimen for male participants and combined NAAT results from clinician-collected vaginal swabs for female participants. The results from NAAT1 and NAAT2 determined if NAAT3 needed to be performed. CRS = composite reference standard. CT = *Chlamydia trachomatis*. MG = *Mycoplasma genitalium*. *N* = total number of evaluable participants. NAAT1 = Cobas 68/8800 CT/NG. NAAT2 = AC2 CT/NG. NAAT3 = Xpert CT/NG. NG = *Neisseria gonorrhoeae*. VS = vaginal swab. VS-C = clinician-collected vaginal swab. VS-S = self-collected vaginal swab.

^*b*^ “Female VS” characteristics are a combination of the “Female VS-C” and “Female VS-S” data presented earlier in the table.

**TABLE 3:** Sensitivity and specificity of the Cobas Liat CT/NG/MG nucleic acid test for detection of NG by specimen type and symptom status (prospectively collected/archived specimen)^*a*^

Specimen type	Symptom status	Total (*N*)	Positive CRS	NG sensitivity		NG specificity	
			% (*n*/*N*)	% (*n*/*N*)	95%	% (*n*/*N*)	95%
Male urine	Symptomatic	938	15.5 (145/938)	100 (145/145)	(97.4, 100)	100 (793/793)	(99.5, 100)
	Asymptomatic	1,526	1.0 (16/1,526)	100 (16/16)	(80.6, 100)	99.8 (1,507/1,510)	(99.4, 99.9)
	Overall	2,464	6.5 (161/2,464)	100 (161/161)	(97.7, 100)	99.9 (2,300/2,303)	(99.6, 100)
Female VS-C	Symptomatic	577	3.3 (19/577)	94.7 (18/19)	(75.4, 99.1)	99.8 (557/558)	(99.0, 100)
	Asymptomatic	714	3.4 (24/714)	100 (24/24)	(86.2, 100)	99.9 (689/690)	(99.2, 100)
	Overall	1,291	3.3 (43/1,291)	97.7 (42/43)	(87.9, 99.6)	99.8 (1,246/1,248)	(99.4, 100)
Female VS-S	Symptomatic	580	4.3 (25/580)	96.0 (24/25)	(80.5, 99.3)	99.8 (554/555)	(99.0, 100)
	Asymptomatic	691	2.7 (19/691)	100 (19/19)	(83.2, 100)	99.9 (671/672)	(99.2, 100)
	Overall	1,271	3.5 (44/1,271)	97.7 (43/44)	(88.2, 99.6)	99.8 (1,225/1,227)	(99.4, 100)
Female VS**^*b*^**	Symptomatic	1,157	3.8 (44/1,157)	95.5 (42/44)	(84.9, 98.7)	99.8 (1,111/1,113)	(99.3, 100)
	Asymptomatic	1,405	3.1 (43/1,405)	100 (43/43)	(91.8, 100)	99.9 (1,360/1,362)	(99.5, 100)
	Overall	2,562	3.4 (87/2,562)	97.7 (85/87)	(92.0, 99.4)	99.8 (2,471/2,475)	(99.6, 99.9)
Female urine	Symptomatic	1,157	4.0 (46/1,157)	89.1 (41/46)	(77.0, 95.3)	99.9 (1,110/1,111)	(99.5, 100)
	Asymptomatic	1,402	2.9 (41/1,402)	97.6 (40/41)	(87.4, 99.6)	99.9 (1,360/1,361)	(99.6, 100)
	Overall	2,559	3.4 (87/2,559)	93.1 (81/87)	(85.8, 96.8)	99.9 (2,470/2,472)	(99.7, 100)

^*a*^ Positive or negative CRS results were derived from combined results from the reference NAATs; these included NAAT results from urine specimen for male participants and combined NAAT results from clinician-collected vaginal swabs for female participants. The results from NAAT1 and NAAT2 determined if NAAT3 needed to be performed. CRS = composite reference standard. CT = *Chlamydia trachomatis*. MG = *Mycoplasma genitalium*. *N* = total number of evaluable participants. NAAT1 = Cobas 68/8800 CT/NG. NAAT2 = AC2 CT/NG. NAAT3 = Xpert CT/NG. NG = *Neisseria gonorrhoeae*. VS = vaginal swab. VS-C = clinician-collected vaginal swab. VS-S = self-collected vaginal swab.

^*b*^ “Female VS” characteristics are a combination of the “Female VS-C” and “Female VS-S” data presented earlier in the table.

**TABLE 4:** Sensitivity and specificity of the Cobas Liat CT/NG/MG nucleic acid test for detection of MG by specimen type and symptom status (prospectively collected specimen)^*a*^

Specimen type	Symptom status	Total (*N*)	Positive CRS	MG sensitivity		MG specificity	
			% (*n*/*N*)	% (*n*/*N*)	95%	% (*n*/*N*)	95%
Male urine	Symptomatic	811	12.3 (100/811)	98.0 (98/100)	(93.0, 99.4)	98.7 (702/711)	(97.6, 99.3)
	Asymptomatic	1,487	7.3 (108/1,487)	96.3 (104/108)	(90.9, 98.6)	99.5 (1,372/1,379)	(99.0, 99.8)
	Overall	2,298	9.1 (208/2,298)	97.1 (202/208)	(93.9, 98.7)	99.2 (2,074/2,090)	(98.8, 99.5)
Female VS-C	Symptomatic	553	12.1 (67/553)	92.5 (62/67)	(83.7, 96.8)	97.7 (475/486)	(96.0, 98.7)
	Asymptomatic	686	6.9 (47/686)	100 (47/47)	(92.4, 100)	98.9 (632/639)	(97.8, 99.5)
	Overall	1,239	9.2 (114/1,239)	95.6 (109/114)	(90.1, 98.1)	98.4 (1,107/1,125)	(97.5, 99.0)
Female VS-S	Symptomatic	563	10.5 (59/563)	98.3 (58/59)	(91.0, 99.7)	96.8 (488/504)	(94.9, 98.0)
	Asymptomatic	670	11.8 (79/670)	92.4 (73/79)	(84.4, 96.5)	97.5 (576/591)	(95.9, 98.5)
	Overall	1,233	11.2 (138/1,233)	94.9 (131/138)	(89.9, 97.5)	97.2 (1,064/1,095)	(96.0, 98.0)
Female VS^*b*^	Symptomatic	1,116	11.3 (126/1,116)	95.2 (120/126)	(90.0, 97.8)	97.3 (963/990)	(96.1, 98.1)
	Asymptomatic	1,356	9.3 (126/1,356)	95.2 (120/126)	(90.0, 97.8)	98.2 (1,208/1,230)	(97.3, 98.8)
	Overall	2,472	10.2 (252/2,472)	95.2 (240/252)	(91.9, 97.3)	97.8 (2,171/2,220)	(97.1, 98.3)
Female urine	Symptomatic	1,114	11.3 (126/1,114)	77.0 (97/126)	(68.9, 83.5)	97.7 (965/988)	(96.5, 98.4)
	Asymptomatic	1,352	9.2 (125/1,352)	80.8 (101/125)	(73.0, 86.7)	99.0 (1,215/1,227)	(98.3, 99.4)
	Overall	2,466	10.2 (251/2,466)	78.9 (198/251)	(73.4, 83.5)	98.4 (2,180/2,215)	(97.8, 98.9)

^*a*^ Positive or negative CRS results were derived from combined results from the reference NAATs; these included NAAT results from urine specimen for male participants and combined NAAT results from clinician-collected vaginal swabs for female participants. The results from NAAT1 and NAAT2 determined if NAAT3 needed to be performed. CRS = composite reference standard. CT = *Chlamydia trachomatis*. MG = *Mycoplasma genitalium*. *N* = total number of evaluable participants. NAAT1 = Cobas 68/8800 CT/NG. NAAT2 = AC2 CT/NG. NAAT3 = Xpert CT/NG. NG = *Neisseria gonorrhoeae*. VS = vaginal swab. VS-C = clinician-collected vaginal swab. VS-S = self-collected vaginal swab.

^*b*^ “Female VS” characteristics are a combination of the “Female VS-C” and “Female VS-S” data presented earlier in the table.

### Clinical performance

Sensitivity estimates for male urine and female vaginal swab specimen were ≥96.4%, ≥91.7%, and ≥95.2% for CT, NG, and MG, respectively; sensitivity estimates were lower using female urine, with values of ≥86.9%, ≥83.3%, and ≥77.0%, for CT, NG, and MG, respectively (Table 5). Across specimen types, specificities were ≥99.7% for CT and NG and ≥97.3% for MG (Table 5). Estimates of sensitivity and specificity for all three pathogens were generally consistent regardless of symptom status, with the exception of results for MG using female urine, which had a significantly lower specificity in symptomatic compared with asymptomatic populations (97.7% vs 99.0%, *P* = 0.015; Table 5).

**TABLE 5:** Comparison of the Cobas Liat CT/NG/MG nucleic acid test performance between symptomatic and asymptomatic population by specimen type for detection of CT, NG, and MG^*a*^

Specimen type	Performance	Symptomatic population % (*n*/*N*)	Asymptomatic population % (*n*/*N*)	Difference (95% CI)^*b*^	*P*-value^*c*^
CT					
Male urine	Sensitivity	98.2 (55/56)	96.4 (53/55)	1.9 (−6.3, 10.8)	0.618
	Specificity	99.9 (751/752)	99.9 (1,432/1,433)	−0.1 (−0.7, 0.3)	1.000
Female VS^*d*^	Sensitivity	98.4 (60/61)	97.9 (47/48)	0.4 (−6.9, 9.5)	1.000
	Specificity	99.7 (1,052/1,055)	99.8 (1,307/1,309)	−0.1 (−0.7, 0.3)	0.662
Female urine	Sensitivity	86.9 (53/61)	87.2 (41/47)	−0.3 (−13.3, 13.8)	1.000
	Specificity	99.8 (1,053/1,055)	99.8 (1,304/1,307)	0.0 (−0.5, 0.5)	1.000
NG					
Male urine	Sensitivity	100 (68/68)	100 (11/11)		
	Specificity	100 (745/745)	99.8 (1,474/1,477)	0.2 (−0.3, 0.6)	0.555
Female VS^*d*^	Sensitivity	91.7 (22/24)	100 (18/18)	−8.3 (−26.1, 10.3)	0.498
	Specificity	99.8 (1,089/1,091)	99.9 (1,337/1,339)	0.0 (−0.5, 0.4)	1.000
Female urine	Sensitivity	83.3 (20/24)	94.4 (17/18)	−11.1 (−31.8, 11.8)	0.371
	Specificity	99.9 (1,089/1,090)	99.9 (1,336/1,337)	0.0 (−0.4, 0.3)	1.000
MG					
Male urine	Sensitivity	98.0 (98/100)	96.3 (104/108)	1.7 (−3.7, 7.4)	0.684
	Specificity	98.7 (702/711)	99.5 (1,372/1,379)	−0.8 (−1.9, 0.0)	0.068
Female VS^*d*^	Sensitivity	95.2 (120/126)	95.2 (120/126)	0.0 (−5.9, 5.9)	1.000
	Specificity	97.3 (963/990)	98.2 (1,208/1,230)	−0.9 (−2.3, 0.3)	0.147
Female urine	Sensitivity	77.0 (97/126)	80.8 (101/125)	−3.8 (−14.0, 6.4)	0.537
	Specificity	97.7 (965/988)	99.0 (1,215/1,227)	−1.3 (−2.6,−0.3)	0.015

^*a*^ CT = *Chlamydia trachomatis*. MG = *Mycoplasma genitalium*. *N* = total number of evaluable participants. NG = *Neisseria gonorrhoeae*. VS = vaginal swab.

^*b*^ CI for difference in performance (i.e., Symptomatic population minus asymptomatic population).

^*c*^ *P*-value was obtained from Fisher’s exact test.

^*d*^ “Female VS” characteristics are a combination of the “Female VS-C” and “Female VS-S” data.

There was no statistically significant difference (*P* < 0.05) in sensitivity or specificity for detection of CT, NG, or MG between clinician- or self-collected vaginal specimens (Table S5). OPAs of ≥96.8% were observed for all sample types in pairwise agreement analyses between the Cobas Liat CT/NG/MG test and NAAT1/NAAT2, as shown in Table S6 for CT, for NG, and for MG detection.

### Operator analysis

Operators at all 13 external sites were selected to represent the intended POC users. A total of 66 non-laboratorians took part in Cobas Liat CT/NG/MG study testing. The educational qualifications of the operators ranged from high school/secondary education to advanced degrees (master’s, PhD, MD), and the number of years of experience in their current position ranged from <6 months to 10 years or more. In ease-of-use questionnaires, the majority of operators strongly agreed or agreed that the Cobas Liat CT/NG/MG test was easy to perform and interpret and that the instructions were clear (Fig. S2).

The clinical performance results for the Cobas Liat CT/NG/MG were compared between the non-laboratorians (*n* = 66) and laboratorians (*n* = 23). Laboratorians were defined as operators who already had training or hands-on experience conducting laboratory testing as a trained professional prior to working with this device. Across both groups, the point estimates for sensitivity and specificity of the Cobas Liat CT/NG/MG performance for detecting each analyte from male urine, female urine, and vaginal swabs were ≥71.8% (Table S7). No statistically significant differences were observed between the non-laboratorians and laboratorians, except for the specificity for NG on vaginal swab (*P* = 0.023) (Table S7). However, given the point estimates of the non-laboratorians (100%) and laboratorians (99.5%) and the narrow 95% CI range accounting for the difference in performance (difference = 0.5 [95% CI: 0.2, 1.3]), it is not believed to be a clinically meaningful result. Overall, the Cobas Liat assay performed well, regardless of the type of users observed in this clinical study (Table S7).

## DISCUSSION

In this non-interventional, multicenter study, the clinical performance of the Cobas Liat CT/NG/MG test was evaluated relative to a CRS, as determined using a combination of three FDA-cleared NAATs and one laboratory-developed test, in both symptomatic and asymptomatic patients from geographically diverse sites across the USA. Our evaluation demonstrated that the Cobas Liat CT/NG/MG test has a good clinical performance for CT/NG/MG detection and offers a rapid and easy-to-use test with centralized laboratory test quality at the POC for both self- and clinician-collected samples. As has been found consistently across multiple platforms, female urine samples produce results with lower sensitivity than vaginal swab samples (25). Based on these data, the FDA and the CDC have recommended vaginal swab samples as the optimal specimen type for maximizing the detection of infection.

The Cobas Liat CT/NG/MG test performed similarly for clinical sensitivity and specificity in symptomatic and asymptomatic individuals and, thus, will serve as an important tool to diagnose and screen for these STIs at the POC. Sensitivity and specificity are recognized to be key performance characteristics of a POC test (20). The Cobas Liat CT/NG/MG test can be performed in a laboratory or in a non-laboratory POC setting, allowing rapid (approximately 20 min) detection of CT, NG, and MG infections in many use-case scenarios. Many clinics do not have access to rapid STI testing and results (16), so this assay addresses a critical unmet need for detecting these relevant pathogens. In the current study, specimen collection took place at 13 geographically diverse clinical sites across the USA, and comprised non-laboratory POC settings, including emergency departments, STI clinics, obstetrics/gynecology offices, and family planning clinics. The ability to perform this test at the POC is a major benefit, as rapid and accurate detection of these STIs within a single patient visit can reduce the need for patient follow-up; ensure timely, accurate treatment; reduce disease transmission; and reduce inappropriate antibiotic prescribing (13, 14, 20, 21). The test represents an opportunity to improve antimicrobial stewardship. This has previously been established in a UK study that quantitatively assessed the impact of rapid testing for CT and NG; the study also showed cost benefits for the clinic-based rapid testing (26). The Cobas Liat test is the first POC testing platform that includes MG, for which symptomatic patients should be tested, according to European Guidelines (11). However, recognizing that guidelines, recommendations, and practices vary across regions, countries, and jurisdictions, Roche has developed a CT/NG only version (Cobas Liat CT/NG test) that does not generate MG results.

Across specimen types and regardless of symptom status, the sensitivity and specificity for all analytes (CT, NG, and MG) was generally high, and similar performances were observed for both self- and clinician-collected vaginal swab samples. As expected, the sensitivity of the Cobas Liat CT/NG/MG test was not as high when using female urine specimens compared with vaginal swabs, which are acknowledged as the optimal sample type for STI testing among women (25, 27). Nonetheless, the option of using female urine as an alternative specimen type may mean the difference between a patient getting STI testing vs no testing at all (25). For instance, for women who are not comfortable with or able to provide a vaginal swab (e.g., as a result of trauma), female urine specimens may serve as an important backup option to the recommended vaginal swab sampling. Indeed, self-collection of specimens has been shown to increase uptake of screening for STIs in vulnerable populations (28). This has onward implications, allowing for more targeted as opposed to empirical treatment decisions.

The Cobas Liat CT/NG/MG test scored well in the ease-of-use questionnaires completed by operators who received no formal training on the assay and who were selected as representative of typical POC operators (e.g., nurses and medical assistants). In addition to clinical sensitivity and specificity, ease of use is a key factor for a POC test (20).

This study had several limitations, including the use of some archived samples for NG testing. The mean (37.9 years) and median (35.0 years) ages of participants in the current study were generally older than those reported in previous studies (5, 7, 8, 23, 26). Future studies will be required to investigate extra-genital specimens (e.g., rectal and oropharyngeal specimens) as the STIs being tested also present in non-urogenital samples (28). The current test is unable to assess the presence of gene mutations that can confer antibiotic resistance, especially for NG and MG, at the time of STI diagnosis. The Cobas Liat CT/NG/MG test has the potential to benefit under-represented communities, such as populations identifying outside the gender binary, as the ability to test self-collected specimen at POC offers the potential to improve access for groups such as these who frequently encounter obstacles in accessing provider testing (29), and for whom the POC may represent a preferred (safer) space for testing. For instance, barriers to testing for gay, bisexual, and other men who have sex with men include fear of the consequences of a positive test, social stigma, distrustful patient–provider relationships, lack of discretion in services, accessibility of health services, and perceptions of risk (29). POC testing may also benefit groups that are disproportionately affected by STIs, including young people; racial and ethnic minority groups; and gay; bisexual, or other men who have sex with men (30). Although the study population included individuals identifying as transmasculine, transfeminine, non-binary, and other gender minorities, the numbers enrolled were insufficient to perform analysis regarding the performance of the test in these populations.

### Conclusions

In this multicenter, clinical performance evaluation study, the Cobas Liat CT/NG/MG test demonstrated good clinical performance, with high sensitivity and specificity for CT, NG, and MG detection in both symptomatic and asymptomatic individuals. The test is rapid and easy to use with centralized testing laboratory quality at the POC for both self- and clinician-collected samples. The overall results of this clinical study support the use of the Cobas Liat CT/NG/MG test for the rapid detection of CT, NG, and MG infections during a single patient visit.

## Data Availability

The raw data supporting the conclusions of this article as well as redacted clinical study documentation such as the clinical study protocol and report will be made available once regulatory review has been completed, dependent on legal agreements between the sponsor, study sites, and participants. Such requests may be directed to rotkreuz.datasharingrequests@roche.com for consideration.

## SUPPLEMENTAL MATERIAL

Figures S1 and S2, and Tables S1 to S7 jcm.00706-25-s0001.docx Fig. S1: Participating clinical study sites. Fig. S2: Operators' responses to ease-of-use questionnaires. Table S1: Exclusion criteria. Table S2: NAATs used for determining CRS. Table S3: Determination of composite comparator status for CT, NG, and MG. Table S4: Subject and sample disposition. Table S5: Comparison of the Cobas Liat CT/NG/MG nucleic acid test performance between VS-C and VS-S by STI. Table S6: Agreement between the Cobas Liat CT/NG/MG nucleic acid test and NAAT1 and NAAT2 tests, respectively, by specimen type for detection of CT NG, and MG. Table S7: Comparison of the clinical performance results for the Cobas Liat CT/NG/MG between non-laboratorians (*n* = 66) and laboratorians (*n* = 23) for CT, NG, and MG.

ASM does not own the copyrights to Supplemental Material that may be linked to, or accessed through, an article. The authors have granted ASM a non-exclusive, world-wide license to publish the Supplemental Material files. Please contact the corresponding author directly for reuse.
