# Supplementary material for: Sensitivity and specificity of the Cobas Liat CT/NG/MG nucleic acid test in a clinical laboratory setting and point-of-care location
Source: J Clin Microbiol. 2025 Oct 29;63(12):e00706-25. doi: 10.1128/jcm.00706-25 (PMC12710341; doi:10.1128/jcm.00706-25)
Supplement: Figures S1 and S2, and Tables S1 to S7 — Fig. S1: Participating clinical study sites. Fig. S2: Operators' responses to ease-of-use questionnaires. Table S1: Exclusion criteria. Table S2: NAATs used for determining CRS. Table S3: Determination of composite comparator status for CT, NG, and MG. Table S4: Subject and sample disposition. Table S5: Comparison of the Cobas Liat CT/NG/MG nucleic acid test performance between VS-C and VS-S by STI. Table S6: Agreement between the Cobas Liat CT/NG/MG nucleic acid test and NAAT1 and NAAT2 tests, respectively, by specimen type for detection of CT NG, and MG. Table S7: Comparison of the clinical performance results for the Cobas Liat CT/NG/MG between non-laboratorians (n = 66) and laboratorians (n = 23) for CT, NG, and MG. [file jcm.00706-25-s0001.docx]

**Title:** Sensitivity and specificity of the Cobas Liat CT/NG/MG nucleic acid test in a clinical laboratory setting and point-of-care location

**Authors:**

Van Der Pol B, PhD, MPH;^1^ Arcenas R, PhD;^2^ Boraas C, MD;^3^ Chavoustie S, MD;^4^ Crane LL, DNP;^5^ d'Empaire N;^6^ Ermel AC, MD;^7^ Harnett G, MD;^8^ Hinestrosa F, MD;^9^ House S, MD, PhD;^10^ Lillis RA, MD;^11^ Miller J, MD;^12^ Mills A, MD;^13^ Poblete R, MD;^14^ Young SA, PhD;^15^ on behalf of the Liat CT/NG/MG Clinical Trial Study Group

**Affiliations:**

^1^University of Alabama at Birmingham Heersink School of Medicine, Birmingham, AL, USA; ^2^Roche Molecular Systems, Pleasanton, CA, USA; ^3^Planned Parenthood North Central States, Saint Paul, MN, USA; ^4^Segal Trials North Miami Office, North Miami, FL, USA; ^5^Planned Parenthood Gulf Coast, Houston, TX, USA; ^6^BioCollections Worldwide, Inc., Miami, FL, USA; ^7^Indiana University School of Medicine, Indianapolis, IN, USA; ^8^No Resistance Consulting Group, LLC, Birmingham, AL, USA; ^9^Orlando Immunology Center, FL, USA; ^10^Washington University School of Medicine, St. Louis, MO, USA; ^11^Henry Ford Hospital, Detroit, MI, USA; ^12^Mills Clinical Research, Los Angeles, CA, USA; ^13^North Jersey Community Research Initiative, Newark, NJ, USA; ^14^TriCore Reference Laboratories, Albuquerque, NM, USA; ^15^Louisiana State University Health Sciences Center, New Orleans, LA, USA.

**Supplementary appendix**

**Fig. A1.** Participating clinical study sites


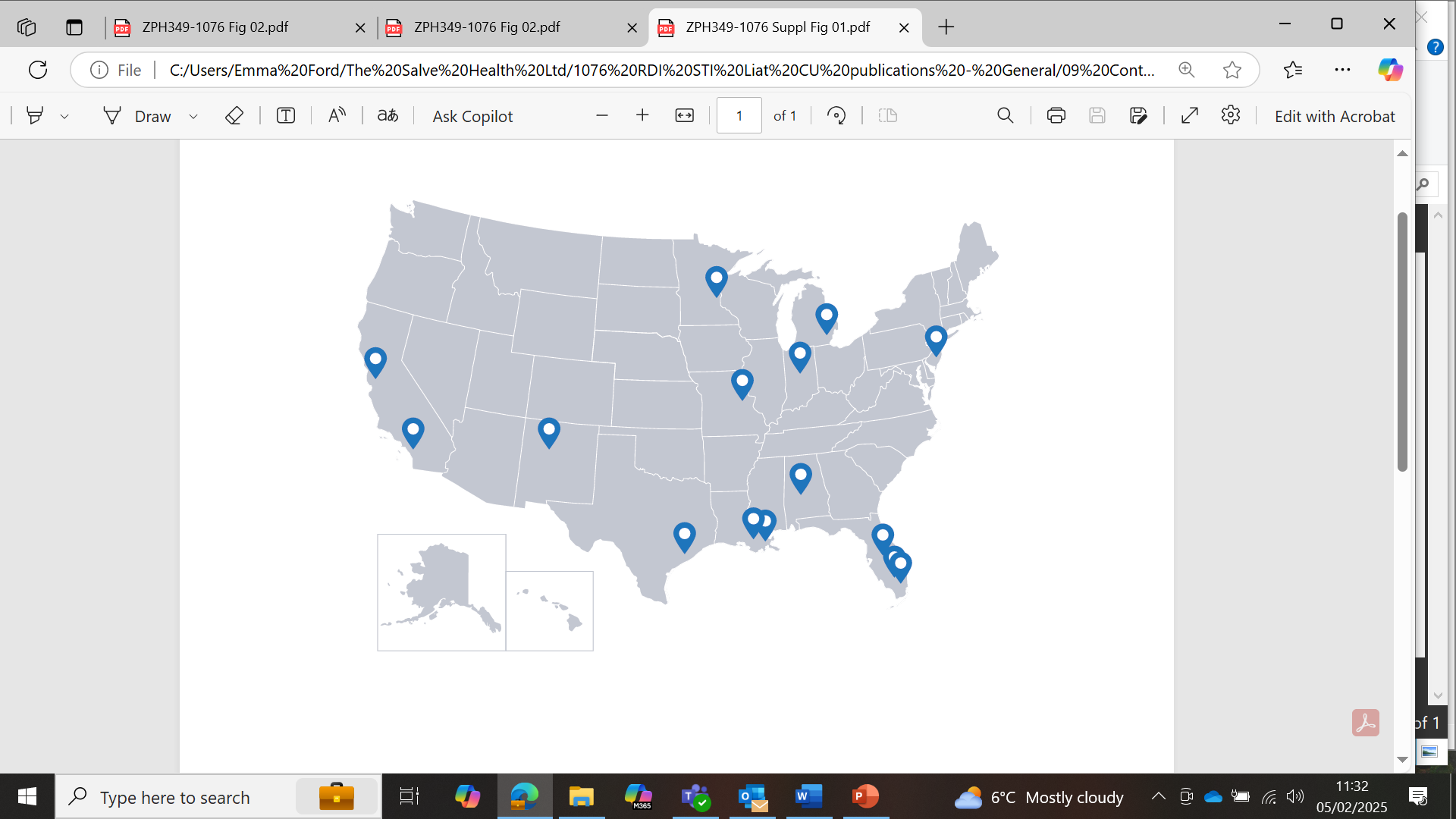


**Fig. A2.** Operators’ responses to ease-of-use questionnaires


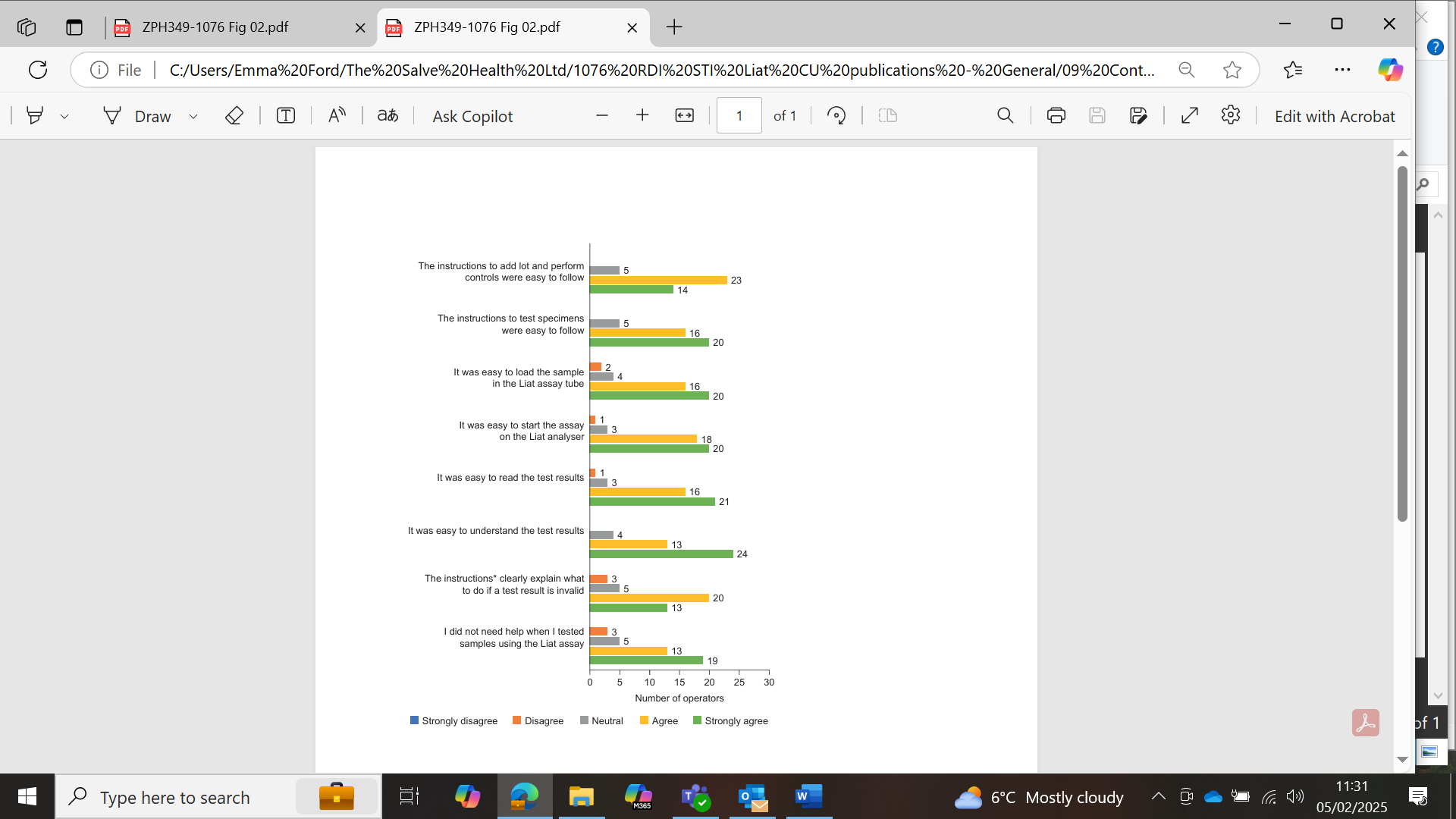


Statements were scored as follows: 1 = strongly disagree; 2 = disagree; 3 = neutral; 4 = agree; 5 = strongly agree. *Included Instructions for Use and Quick Reference Instructions.

**Table A1.** Exclusion criteria

| Patients, regardless of symptom status, were excluded from enrolment if they met any of the following criteria:   - Previously enrolled in the study - Use of antimicrobial agents active against CT, NG, or MG during the 21 days prior to sample collection. Example of such antimicrobial agents include:   - Macrolides (eg, azithromycin, erythromycin)   - Penicillins (eg, amoxicillin)   - Tetracyclines (eg, doxycycline)   - Fluoroquinolones (eg, ciprofloxacin, ofloxacin, levofloxacin), or   - Cephalosporins (eg, ceftriaxone, cefixime) - Use of any over-the-counter feminine hygiene products (internally or externally), such as vaginal moisturizers, lubricants (eg, Replens, RepHresh), and feminine washes/vaginal douches, within the 3 days prior to sample collection. The use of tampons or pads during menses should not be considered exclusionary criteria. - Contraindication to vaginal swab sampling - Urination within 1 hour prior to sample collection |
| --- |

CT=*Chlamydia trachomatis*. MG=*Mycoplasma genitalium*. NG=*Neisseria gonorrhoeae*.

**Table A2.** NAATs used for determining CRS

| **Purpose** | **Assays used** | **Collection tube** |
| --- | --- | --- |
| CT and NG testing | NAAT 1: HOLOGIC^®^ Aptima^®^ Combo 2^®^ Assay (for CT and NG) (AC2 CT/NG; Hologic, Inc., San Diego, CA) | Aptima collection device |
|  | NAAT 2: Cobas^®^ CT/NG Nucleic Acid Test for use on the Cobas 6800/8800 System (Cobas 68/8800 CT/NG; Roche Molecular Systems, Inc.) | Cobas PCR media collection device |
|  | NAAT 3 (tie-breaker test, as needed): Cepheid^®^ Xpert^®^ CT/NG (Cepheid, Sunnyvale, CA) | Xpert collection device |
| MG testing | NAAT 1: HOLOGIC Aptima^®^ *Mycoplasma genitalium* Assay (Aptima MG; Hologic, Inc., San Diego, CA) | Aptima collection device |
|  | NAAT 2: Cobas TV/MG Nucleic Acid Test for use on the Cobas 6800/8800 Systems (Cobas 68/8800 TV/MG; Roche Molecular Systems, Inc.) | Cobas PCR media collection device |
|  | NAAT 3 (tie-breaker test, as needed): Alternative In-House Developed Assay (AIDA) for MG, Nucleic Acid Test (PCR) for the detection of MG (AIDA MG; Roche Molecular Systems, Inc.) | Cobas PCR media collection device |

CRS=composite reference standard. CT=*Chlamydia trachomatis*. MG*=Mycoplasma genitalium*. NAAT=nucleic acid amplification test. NG*=Neisseria gonorrhoeae.* PCR=*polymerase chain reaction*.

**Table A3.** Determination of composite comparator status for CT, NG, and MG

| **NAAT1** | **NAAT2** | **NAAT3  (if needed)** | **CRS** |
| --- | --- | --- | --- |
| + | + | n/a | Positive |
| + | − | + | Positive |
| − | + | + | Positive |
| − | − | n/a | Negative |
| + | − | − | Negative |
| − | + | − | Negative |
| − | Invalid | + | Indeterminate |
| − | Invalid | − | Negative |
| Invalid | − | + | Indeterminate |
| Invalid | − | − | Negative |
| + | Invalid | − | Indeterminate |
| Invalid | + | − | Indeterminate |
| + | Invalid | + | Positive |
| Invalid | + | + | Positive |
| Invalid | Invalid | n/a | Indeterminate |

The CRS designations of “positive” or “negative” for each individual analyte (CT, NG, and MG) were based on combined results from three Food and Drug Administration-cleared NAATs and one laboratory-developed test. The results from NAAT1 and NAAT2 determined if NAAT3 needed to be performed.
CRS=composite reference standard. CT=*Chlamydia trachomatis*. MG=*Mycoplasma genitalium*. n/a=not applicable. NAAT=nucleic acid amplification test. NG=*Neisseria gonorrhoeae*.

**Table A4.** Subject and sample disposition

|  | **Number of participants** | | | |
| --- | --- | --- | --- | --- |
|  | **Total** | **CT** | **NG** | **MG** |
| Total screened | 4,858 | 4,858 | 4,858 | 4,858 |
| Excluded* | 6 | 6 | 6 | 6 |
| Protocol deviation | 4 | 4 | 4 | 4 |
| Withdrawal by subject | 1 | 1 | 1 | 1 |
| Other | 1 | 1 | 1 | 1 |
| Included (enrolled) | 4,852 | 4,852 | 4,852 | 4,852 |
| Sex assigned at birth |  |  |  |  |
| Male | 2,340 | 2,340 | 2,340 | 2,340 |
| Female | 2,512 | 2,512 | 2,512 | 2,512 |
| Evaluable^†^ | 4,800 | 4,790 | 4,793 | 4,791 |
| Male urine | 2,309 | 2,301 | 2,306 | 2,303 |
| Female VS-C | 1,244 | 1,244 | 1,243 | 1,243 |
| Female VS-S | 1,237 | 1,236 | 1,235 | 1,235 |
| Female urine | 2,478 | 2,474 | 2,473 | 2,472 |
| Non-evaluable^‡^ | 52 | 62 | 59 | 61 |
| Uninterpretable CRS | 30 | 35 | 35 | 34 |
| Missing/invalid Cobas Liat CT/NG/MG test results | 13 | 18 | 15 | 18 |
| Protocol deviation/incident reported | 9 | 9 | 9 | 9 |

*Participants who did not meet the study criteria were excluded from all analyses. ^†^Participants with a positive or negative composite reference standard (CRS) and a valid test result with the Cobas Liat CT/NG/MG nucleic acid test were considered evaluable and included in the final analysis. ^‡^Participants contributing invalid results with the Cobas Liat CT/NG/MG test across all specimen types and/or with a CRS of “Uninterpretable” were considered non-evaluable.
CT=*Chlamydia trachomatis*. MG=*Mycoplasma genitalium*. NG=*Neisseria gonorrhoeae*. VS-C=clinician-collected vaginal swab. VS-S=self-collected vaginal swab.

**Table A5** Comparison of the Cobas Liat CT/NG/MG nucleic acid test performance between VS-C and VS-S by STI

| **Target** | **Performance** | **Clinician-collected vaginal specimens  % (n/N)** | **Self-collected vaginal specimens  % (n/N)** | **Difference (95% CI)*** | **p-value**^†^ |
| --- | --- | --- | --- | --- | --- |
| CT | Sensitivity | 96.0 (48/50) | 100 (59/59) | −4.0 (−13.5, 2.3) | 0.208 |
|  | Specificity | 99.8 (1,187/1,189) | 99.7 (1,172/1,175) | 0.1 (−0.4, 0.6) | 0.685 |
| NG | Sensitivity | 94.7 (18/19) | 95.7 (22/23) | −0.9 (−21.3, 16.9) | 1.000 |
|  | Specificity | 99.8 (1,217/1,219) | 99.8 (1,209/1,211) | 0.0 (−0.4, 0.5) | 1.000 |
| MG | Sensitivity | 95.6 (109/114) | 94.9 (131/138) | 0.7 (−5.4, 6.4) | 1.000 |
|  | Specificity | 98.4 (1,107/1,125) | 97.2 (1,064/1,095) | 1.2 (0.0, 2.5) | 0.060 |

*CI for difference in performance (ie VS-C minus VS-S). ^†^p-value was obtained from Fisher’s exact test.

CT=*Chlamydia trachomatis.* MG=*Mycoplasma genitalium*. N=total number of evaluable participants. NG=*Neisseria gonorrhoeae*. STI=sexually transmitted infection. VS-C=clinician-collected vaginal swab. VS-S=self-collected vaginal swab.

**Table A6.** Agreement between the Cobas Liat CT/NG/MG nucleic acid test and NAAT1 and NAAT2 tests, respectively, by specimen type for detection of CT NG, and MG

| **Detection of CT** | | | | |
| --- | --- | --- | --- | --- |
| **Specimen type** | **Cobas Liat CT/NG/MG test result** | **NAAT 1 CT positive** | **NAAT 1 CT negative** | **Total** |
| Male urine | CT detected | 109 | 2 | 111 |
|  | CT not detected | 5 | 2,184 | 2,189 |
|  | Total | 114 | 2,186 | 2,300 |
|  | Agreement (95% CI) | PPA: 95.6% (90.1, 98.1) | NPA: 99.9% (99.7, 100) | OPA: 99.7% (99.4, 99.9) |
| Female VS-C | CT detected | 48 | 2 | 50 |
|  | CT not detected | 2 | 1,183 | 1,185 |
|  | Total | 50 | 1,185 | 1,235 |
|  | Agreement (95% CI) | PPA: 96.0% (86.5, 98.9) | NPA: 99.8% (99.4,100) | OPA: 99.7% (99.2, 99.9) |
| Female VS-S | CT detected | 59 | 3 | 62 |
|  | CT not detected | 0 | 1,171 | 1,171 |
|  | Total | 59 | 1,174 | 1,233 |
|  | Agreement (95% CI) | PPA: 100 (93.9, 100) | NPA: 99.7% (99.3, 99.9) | OPA: 99.8% (99.3, 99.9) |
| Female urine | CT detected | 94 | 3 | 97 |
|  | CT not detected | 6 | 2,372 | 2,378 |
|  | Total | 100 | 2,375 | 2,475 |
|  | Agreement (95% CI) | PPA: 94.0% (87.5, 97.2) | NPA: 99.9% (99.6, 100) | OPA: 99.6% (99.3, 99.8) |
|  |  | **NAAT 2 CT positive** | **NAAT 2 CT negative** |  |
| Male urine | CT detected | 107 | 3 | 110 |
|  | CT not detected | 3 | 2,184 | 2,187 |
|  | Total | 110 | 2,187 | 2,997 |
|  | Agreement (95% CI) | PPA: 97.3% (92.3, 99.1) | NPA: 99.9% (99.6, 100) | OPA: 99.7 (99.4, 99.9) |
| Female VS-C | CT detected | 49 | 1 | 50 |
|  | CT not detected | 8 | 1,179 | 1,187 |
|  | Total | 57 | 1,180 | 1,237 |
|  | Agreement (95% CI) | PPA: 86.0% (74.7, 92.7) | NPA: 99.9% (99.5, 100) | OPA: 99.3% (98.6, 99.6) |
| Female VS-S | CT detected | 59 | 4 | 63 |
|  | CT not detected | 1 | 1,172 | 1,173 |
|  | Total | 60 | 1,176 | 1,236 |
|  | Agreement (95% CI) | PPA: 98.3% (91.1, 99.7) | NPA: 99.7% (99.1, 99.9) | OPA: 99.6% (99.1, 99.8) |
| Female urine | CT detected | 91 | 8 | 99 |
|  | CT not detected | 9 | 2,360 | 2,369 |
|  | Total | 100 | 2,368 | 2,468 |
|  | Agreement (95% CI) | PPA: 91.0% (83.8, 95.2) | NPA: 99.7% (99.3, 99.8) | OPA: 99.3% (98.9, 99.6) |
| **Detection of NG** | | | | |
|  | **Cobas Liat CT/NG/MG test result** | **NAAT 1 NG positive** | **NAAT 1 NG negative** |  |
| Male urine | NG detected | 79 | 3 | 82 |
|  | NG not detected | 3 | 2,218 | 2,221 |
|  | Total | 82 | 2,221 | 2,303 |
|  | Agreement (95% CI) | PPA: 96.3% (89.8, 98.7) | NPA: 99.9% (99.6, 100) | OPA: 99.7% (99.4, 99.9) |
| Female VS-C | NG detected | 18 | 2 | 20 |
|  | NG not detected | 3 | 1,212 | 1,215 |
|  | Total | 21 | 1,214 | 1,235 |
|  | Agreement (95% CI) | PPA: 85.7% (65.4, 95.0) | NPA: 99.8% (99.4, 100) | OPA: 99.6% (99.1, 99.8) |
| Female VS-S | NG detected | 22 | 2 | 24 |
|  | NG not detected | 1 | 1,209 | 1,210 |
|  | Total | 23 | 1,211 | 1,234 |
|  | Agreement (95% CI) | PPA: 95.7% (79.0, 99.2) | NPA: 99.8% (99.4, 100) | OPA: 99.8% (99.3, 99.9) |
| Female urine | NG detected | 37 | 2 | 39 |
|  | NG not detected | 2 | 2,436 | 2,438 |
|  | Total | 39 | 2,438 | 2,477 |
|  | Agreement (95% CI) | PPA: 94.9% (83.1, 98.6) | NPA: 99.9% (99.7, 100) | OPA: 99.8% (99.6, 99.9) |
|  |  | **NAAT 2 NG positive** | **NAAT 2 NG negative** |  |
| Male urine | NG detected | 79 | 3 | 82 |
|  | NG not detected | 0 | 2,219 | 2,219 |
|  | Total | 79 | 2,222 | 2,301 |
|  | Agreement (95% CI) | PPA: 100% (95.4, 100) | NPA: 99.9% (99.6, 100) | OPA: 99.9% (99.6, 100) |
| Female VS-C | NG detected | 16 | 3 | 19 |
|  | NG not detected | 1 | 1,215 | 1,216 |
|  | Total | 17 | 1,218 | 1,235 |
|  | Agreement (95% CI) | PPA: 94.1% (73.0, 99.0) | NPA: 99.8% (99.3, 99.9) | OPA: 99.7% (99.2, 99.9) |
| Female VS-S | NG detected | 19 | 3 | 22 |
|  | NG not detected | 0 | 1,211 | 1,211 |
|  | Total | 19 | 1,214 | 1,233 |
|  | Agreement (95% CI) | PPA: 100% (83.2, 100) | NPA: 99.8% (99.3, 99.9) | OPA: 99.8% (99.3, 99.9) |
| Female urine | NG detected | 35 | 4 | 39 |
|  | NG not detected | 0 | 2,430 | 2,430 |
|  | Total | 35 | 2,434 | 2,469 |
|  | Agreement (95% CI) | PPA: 100 (90.1, 100) | NPA: 99.8% (99.6, 99.9) | OPA: 99.8% (99.6, 99.9) |
| **Detection of MG** | | | | |
|  |  | **NAAT 1 MG positive** | **NAAT 1 MG negative** |  |
| Male urine | MG detected | 204 | 15 | 219 |
|  | MG not detected | 5 | 2,073 | 2,078 |
|  | Total | 209 | 2,088 | 2,297 |
|  | Agreement (95% CI) | PPA: 97.6% (94.5, 99.0) | NPA: 99.3% (98.8, 99.6) | OPA: 99.1% (98.7, 99.4) |
| Female VS-C | MG detected | 100 | 28 | 128 |
|  | MG not detected | 1 | 1,111 | 1,112 |
|  | Total | 101 | 1,139 | 1,240 |
|  | Agreement (95% CI) | PPA: 99.0% (94.6, 99.8) | NPA: 97.5% (96.5, 98.3) | OPA: 97.7% (96.7, 98.4) |
| Female VS-S | MG detected | 124 | 38 | 162 |
|  | MG not detected | 2 | 1,071 | 1,073 |
|  | Total | 126 | 1,109 | 1,235 |
|  | Agreement (95% CI) | PPA: 98.4% (94.4, 99.6) | NPA: 96.6% (95.3, 97.5) | OPA: 96.8% (95.6, 97.6) |
| Female urine | MG detected | 187 | 46 | 233 |
|  | MG not detected | 11 | 2,226 | 2,237 |
|  | Total | 198 | 2,272 | 2,470 |
|  | Agreement (95% CI) | PPA: 94.4% (90.3, 96.9) | NPA: 98.0% (97.3, 98.5) | OPA: 97.7% (97.0, 98.2) |
|  |  | **NAAT 2 MG positive** | **NAAT 2 MG negative** |  |
| Male urine | MG detected | 208 | 9 | 217 |
|  | MG not detected | 12 | 2,064 | 2,076 |
|  | Total | 220 | 2,073 | 2,293 |
|  | Agreement (95% CI) | PPA: 94.5% (90.7, 96.9) | NPA: 99.6% (99.2, 99.8) | OPA: 99.1% (98.6, 99.4) |
| Female VS-C | MG detected | 125 | 1 | 126 |
|  | MG not detected | 14 | 1,090 | 1,104 |
|  | Total | 139 | 1,091 | 1,230 |
|  | Agreement (95% CI) | PPA: 89.9% (83.8, 93.9) | NPA: 99.9% (99.5,100) | OPA: 98.8% (98.0, 99.3) |
| Female VS-S | MG detected | 149 | 13 | 162 |
|  | MG not detected | 17 | 1,051 | 1,068 |
|  | Total | 166 | 1,064 | 1,230 |
|  | Agreement (95% CI) | PPA: 89.8% (84.2, 93.5) | NPA: 98.8% (97.9, 99.3) | OPA: 97.6% (96.5, 98.3) |
| Female urine | MG detected | 215 | 16 | 231 |
|  | MG not detected | 39 | 2,190 | 2,229 |
|  | Total | 254 | 2,206 | 2,460 |
|  | Agreement (95% CI) | PPA: 84.6% (79.7, 88.6) | NPA: 99.3% (98.8, 99.6) | OPA: 97.8% (97.1, 98.3) |

CI=confidence interval. CT=*Chlamydia trachomatis*. MG=*Mycoplasma genitalium*. N=total number of evaluable participants. NAAT1=Cobas 68/8800 CT/NG. NAAT2=AC2 CT/NG. NG=*Neisseria gonorrhoeae.* NPA=negative percentage agreement. OPA=overall percentage agreement. PPA=positive percentage agreement.
VS-C=clinician-collected vaginal swab. VS-S=self-collected vaginal swab.

**Table A7.** Comparison of the clinical performance results for the Cobas Liat CT/NG/MG between non-laboratorians (n=66) and laboratorians (n=23) for CT, NG, and MG

| **Specimen type** | **Performance** | **Laboratorian % (n/N)** | **Non-laboratorian % (n/N)** | **Difference (95% Score CI)*** | **p-value**^†^ |
| --- | --- | --- | --- | --- | --- |
| **CT** | | | | | |
| Male urine | Sensitivity | 97.3 (36/37) | 97.3 (71/73) | 0.0 (−11.4, 7.3) | 1.000 |
|  | Specificity | 100 (774/774) | 99.8 (1,292/1,294) | −0.2% (−0.6, 0.3) | 0.531 |
| Female VS^†^ | Sensitivity | 100 (23/23) | 97.6 (83/85) | −2.4% (−8.2, 12.2) | 1.000 |
|  | Specificity | 99.8 (655/656) | 99.8 (1,620/1,624) | −0.1% (−0.5, 0.6) | 1.000 |
| Female urine | Sensitivity | 86.4 (19/22) | 87.1 (74/85) | −0.7% (−21.4, 12.2) | 1.000 |
|  | Specificity | 99.8 (652/653) | 99.8 (1,624/1,627) | −0.0 (−0.4, 0.7) | 1.000 |
| **NG** | | | | | |
| Male urine | Sensitivity | 100 (34/34) | 100 (42/42) | 0 | - |
|  | Specificity | 99.6 (773/776) | 100 (1,325/1,325) | 0.4 (0.1, 1.1) | 0.050 |
| Female VS^†^ | Sensitivity | 100 (13/13) | 92.9 (26/28) | −7.1 (−22.9, 16.7) | 1.000 |
|  | Specificity | 99.5 (663/666) | 100 (1,679/1,679) | 0.5 (0.2, 1.3) | 0.023 |
| Female urine | Sensitivity | 78.6 (11/14) | 92.9 (26/28) | −14.3 (−41.9, 6.6) | 0.313 |
|  | Specificity | 100 (661/661) | 99.9 (1,679/1,681) | −0.1 (−0.4, 0.5) | 1.000 |
| **MG** | | | | | |
| Male urine | Sensitivity | 97.6 (82/84) | 96.6 (115/119) | 1.0 (−5.3, 6.3) | 1.000 |
|  | Specificity | 99.2 (719/725) | 99.2 (1,237/1,247) | 0.0 (−0.8, 1.1) | 1.000 |
| Female VS^†^ | Sensitivity | 92.3 (72/78) | 97.0 (164/169) | −4.7 (−13.1, 0.8) | 0.106 |
|  | Specificity | 97.2 (582/599) | 97.9 (1,507/1,539) | 0.8 (−0.6, 2.5) | 0.334 |
| Female urine | Sensitivity | 71.8 (56/78) | 82.2 (139/169) | −10.5 (−22.5, 0.5) | 0.067 |
|  | Specificity | 98.7 (586/594) | 98.3 (1,514/1,540) | −0.3 (−1.4, 1.0) | 0.701 |

*CI for difference in performance (ie non-laboratorian minus laboratorian); ^†^p-value was obtained from Fisher’s exact test. ^†^“Female VS” characteristics are a combination of the “Female VS-C” and “Female VS-S” data.
CI=confidence interval. CT=*Chlamydia trachomatis*. MG=*Mycoplasma genitalium*. N=total number of evaluable participants. NG=*Neisseria gonorrhoeae.* VS=vaginal swab.
